# Prevalence and Magnitude of Potential Surprise Bills for Childbirth

**DOI:** 10.1001/jamahealthforum.2021.1460

**Published:** 2021-07-02

**Authors:** Kao-Ping Chua, A. Mark Fendrick, Rena M. Conti, Michelle H. Moniz

**Affiliations:** 1 Department of Pediatrics, Susan B. Meister Child Health Evaluation and Research Center, University of Michigan Medical School, Ann Arbor; 2 Department of Health Management and Policy, University of Michigan School of Public Health, Ann Arbor; 3 Department of Internal Medicine, University of Michigan Medical School, Ann Arbor; 4 Center for Value-Based Insurance Design, University of Michigan, Ann Arbor; 5 Department of Markets, Public Policy, and Law, Institute for Health System Innovation and Policy, Questrom School of Business, Boston University, Boston, Massachusetts; 6 Department of Obstetrics and Gynecology, University of Michigan Medical School, Ann Arbor

## Abstract

This cross-sectional study examines surprise bills that are received for childbirths and newborn hospitalizations.

## Introduction

In 2022, federal legislation will protect families from most *surprise bills*,^1^ which are defined as charges for out-of-network-care at in-network facilities.^2,3,4^ To illustrate the potential benefits of this legislation, we estimated the frequency and magnitude of surprise bills for deliveries and newborn hospitalizations, which are the leading reasons for hospitalization in the US.

## Methods

We analyzed 2019 data from Optum’s deidentified Clinformatics Data Mart, which includes 12 million privately insured enrollees in all states.^5^ Analyses included families with an in-network delivery in 2019 that could be linked to 1 or more in-network newborn hospitalization that was covered by the same family plan (eAppendix 1 in the Supplement). We only included 1 delivery per family. Deliveries were linked with 1 newborn hospitalization unless multiple births occurred (eg, twins). As data were deidentified, this study was exempted from human participants review by the institutional review board of the University of Michigan Medical School; informed consent was not required. This study follows the Strengthening the Reporting of Observational Studies in Epidemiology (STROBE) reporting guidelines for cross-sectional studies.

*Potential surprise bills* were defined as professional claims from out-of-network clinicians and ancillary service providers (eg, ambulance providers). We estimated the liability for these bills by subtracting typical in-network reimbursement from charges.^2,3,4^ Among families with 1 or more potential surprise bill for the delivery, newborn hospitalization(s), or both, we calculated the median total liability for potential surprise bills (sum of the liability across the delivery and newborn hospitalization[s]).

To evaluate whether surprise billing protections would be more beneficial for resource-intensive hospitalizations, we assessed the frequency and magnitude of potential surprise bills among deliveries with and without 1 or more claim for cesarean delivery and among newborn hospitalizations with and without 1 or more claim for neonatal intensive care. For deliveries and newborn hospitalizations, we identified which services accounted for the greatest numbers of potential surprise bills. Analyses were conducted using SAS, version 9.4 (SAS Institute).

## Results

Analyses included 95 384 families. Deliveries for these families were linked to 96 881 newborn hospitalizations. Of all families, 17 949 (18.8%) had 1 or more potential surprise bill for the delivery, newborn hospitalization(s), or both. Among these families, the median total liability for potential surprise bills was $744 (25th-75th percentile, $138-$3823); for 6417 families (35.8%), total liability exceeded $2000.

Among 32 203 and 63 181 deliveries with and without 1 or more cesarean delivery claims, 6594 (20.5%) and 5597 (8.9%) had 1 or more potential surprise bill, with a median (25th-75th percentile) liability of $1825 ($272-$5624) and $900 ($124-$3642), respectively (Table 1). For deliveries, the service accounting for the highest share of the 32 837 potential surprise bills was anesthesia for vaginal birth (5369 [16.3%]; Table 2).

**Table 1.** Prevalence and Magnitude of Potential Surprise Bills According to the Occurrence of Cesarean Delivery and Neonatal Intensive Care

Outcome	All deliveries (N = 95 384)^a^	≥1 Cesarean delivery claim (n = 32 203)^b^	No cesarean delivery claims (n = 63 181)^b^	Difference between medians (95% CI)^c^
**Deliveries**				
No potential surprise bills, No. (%)	83 193 (87.2)	25 609 (79.5)	57 854 (91.1)	NA
≥1 Potential surprise bill, No. (%)	12 191 (12.8)	6594 (20.5)	5597 (8.9)	NA
Median estimated liability for potential surprise bills (25th-75th percentile), $^d^	1356 (180-4595)	1825 (272-5624)	900 (124-3642)	925 (760-1091)
**Newborn hospitalizations**	**All newborn hospitalizations (n = 96 881)** ^**a**^	**≥1 Neonatal intensive care claim (n = 5970)** ^**b**^	**No neonatal intensive care claims (n = 90 991)** ^**b**^	**Difference between medians (95% CI)** ^**c**^
No potential surprise bills, episodes, No. (%)	87 854 (90.7)	5044 (84.5)	82 810 (91.1)	NA
≥1 Potential surprise bill, episodes, No. (%)	9027 (9.3)	926 (15.5)	8101 (8.9)	NA
Median estimated liability for potential surprise bills (25th-75th percentile), $^d^	262 (123-766)	1282 (217-10 472)	262 (123-622)	1031 (971-1091)

Abbreviation: NA, not applicable.

^a^ The 95 384 families were linked to 96 881 newborn hospitalizations. Among the 95 384 families, 93 910 had deliveries linked to 1 newborn hospitalization, 1451 had deliveries linked to 2 newborn hospitalizations, and 23 had deliveries linked to 3 newborn hospitalizations. For the latter 2 groups, the multiple newborn hospitalizations were for unique newborns.

^b^ See eAppendix 2 in the Supplement for codes used to define these claims.

^c^ May not equal the difference between columns 2 and 3 because of rounding. We calculated confidence intervals using quantile regression.

^d^ Equals out-of-network charge minus standardized cost, an estimate of the insurer’s national average in-network reimbursement.

**Table 2.** Service Categories Accounting for the 5 Greatest Numbers of Potential Surprise Bills for Deliveries and Newborn Hospitalizations

Service category^a^	No. of claims for potential surprise bills	Claims for potential surprise bills in sample, %	Sum of estimated liability across all potential surprise bills in sample, $	Estimated liability across all potential surprise bills in sample, %	Estimated liability per potential surprise bill, $
**Deliveries**					
Anesthesia: vaginal delivery^b^	5369	16.3	17 461 596	33.5	3252
Obstetrical: cesarean delivery	4691	14.2	23 086 175	44.2	4921
Laboratory: blood transfusion^c^	4365	13.3	68 189	0.1	16
Laboratory: other hematology^d^	3779	11.5	52 955	0.1	14
Anesthesia: cesarean delivery	3301	.0	3 528 432	6.8	1069
All other service categories	11 433	34.7	7 999 481	15.3	700
Total	32 938	100.0	52 196 827	100.0	1585
**Newborn hospitalizations**					
Inpatient: neonatal intensive care	6930	19.4	10 092 679	46.3	1456
Laboratory: other hematology^d^	6086	17.0	103 041	0.5	17
Hearing: complex audiological function tests^e^	5457	15.3	1 593 521	7.3	292
Obstetrical: newborn care^f^	5018	14.1	2 322 768	10.7	463
Inpatient: discharge management	2390	6.7	1 459 635	6.7	611
All other service categories	9834	27.5	6 222 351	28.5	633
Total	35 715	100.0	21 793 995	100.0	610

Abbreviation: CBC, complete blood count.

^a^ Service categories were defined by the database vendor.

^b^ For example, neuraxial labor analgesia/anesthesia (eg, epidural placement).

^c^ For example, ABO blood typing.

^d^ For example, CBC test.

^e^ For example, auditory evoked potentials for evoked response audiometry and/or testing of the central nervous system.

^f^ For example, newborn evaluation and management and delivery/birthing room resuscitation.

Among 5970 and 90 991 newborn hospitalizations with and without 1 or more neonatal intensive care claim, 926 (15.5%) and 8101 (8.9%) had 1 or more potential surprise bill, with a median (25th-75th percentile) liability of $1282 ($217-$10 472) and $262 ($123-$766), respectively (Table 1). For newborn hospitalizations, the service accounting for the highest share of the 35 715 potential surprise bills was neonatal intensive care (6930 [19.4%]; Table 2).

## Discussion

Among privately insured families with in-network deliveries in 2019, almost 1 in 5 potentially received surprise bills for maternal and/or newborn care. For these families, estimated liability for potential surprise bills averaged $744, an amount larger than the estimated liability for colonoscopy but smaller than that for surgical care.^2,3^ For one-third of families that received potential surprise bills, the estimated liability exceeded $2000. Surprise bills were more frequent and larger when cesarean delivery or neonatal intensive care occurred.

Our study was limited by lack of information on whether families received surprise bills or actual amounts paid. Despite this limitation, our findings suggest that federal protections against surprise bills could benefit many families, particularly when resource-intensive hospitalizations occur. Importantly, these protections will not alleviate the substantial costs of childbirth that occur even without surprise bills. In a study of privately insured women, out-of-pocket spending for maternal care between the 12 months before to 3 months after delivery averaged $4500.^6^ While surprise billing protections are important first steps, improvements in childbirth benefit design are needed to protect families from undue financial burden.

Notably, the high frequency of out-of-network care in our study, coupled with the fact that childbirth is the most common reason for hospitalization, suggests that childbirth hospitalizations are currently one of the most frequent sources of surprise bills in the US. Consequently, inadequate enforcement of federal protections could result in many erroneous surprise bills. Policy makers may wish to devote additional resources to enforcement for childbirth hospitalizations.

## Notes

Supplement. eAppendix 1. Details on identifying study sample eAppendix 2. Codes used to identify Cesarean delivery and neonatal intensive care claims
