# Supplementary material for: Prevalence and Magnitude of Potential Surprise Bills for Childbirth
Source: JAMA Health Forum. 2021 Jul 2;2(7):e211460. doi: 10.1001/jamahealthforum.2021.1460 (PMC8796895; doi:10.1001/jamahealthforum.2021.1460)
Supplement: Supplement. — eAppendix 1. Details on identifying study sample eAppendix 2. Codes used to identify Cesarean delivery and neonatal intensive care claims [file jamahealthforum-e211460-s001.pdf]

Supplemental Online Content

Chua K-P, Fendrick AM, Conti RM, Moniz MH. Prevalence and magnitude of potential surprise bills for childbirth. *JAMA Health Forum*. 2021;2(7):e211460. doi:10.1001/jamahealthforum.2021.1460

eAppendix 1. Details on identifying study sample

eAppendix 2. Codes used to identify Cesarean delivery and neonatal intensive care claims

This supplemental material has been provided by the authors to give readers additional information about their work.

© 2021 Chua K-P et al. *JAMA Health Forum.*

eAppendix 1. Details on identifying study sample

"Deliveries" were in-network hospitalizations for females aged 12-55 years that began in 2019 and had ≥1 claim with a birth-related diagnosis, procedure, or revenue code. To identify deliveries, we used a modified list of birth-related diagnosis and procedure codes included in a published algorithm[1], as well as labor and delivery revenue codes.

- Birth-related International Classification of Diseases, Tenth Revision, Clinical Modification (ICD-10-CM) diagnosis codes were O1002, O1012, O1022, O1032, O1042, O1092, O114, O1204, O1214, O1224, O134, O1404, O1414, O1424, O1494, O164, O2402, O2412, O2432, O24420-O24429, O2482, O2492, O252, O2662, O2672, O4202, O4212, O4292, O601xxx-O602xxx, O61xxxx-O82xxxx, O8802, O8812, O8822, O8832, O8882, O9802, O9812, O9822, O9832, O9842, O9852, O9862, O9872, O9882, O9892, O9902, O9912, O99214, O99214, O99284, O99314, O99324, O99334, O99344, O99354, O9942, O9952, O9962, O9972, O99814, O99824, O99834, O99844, O9A12, O9A22, O9A32, O9A42, O9A52, Z37xx-Z38xx (except Z371, Z374, and Z377).
- Current Procedural Terminology (CPT) codes were 01960, 01961, 01962, 01963, 01967, 01968, 01969, 58611, 59300, 59400, 59409, 59410, 59414, 59510, 59514, 59515, 59525, 59610, 59612, 59614, 59618, 59620, 59622.
- ICD-10-CM procedure codes were 0Q820ZZ, 0Q823ZZ, 0Q824ZZ, 0Q830ZZ, 0Q833ZZ, 0Q834ZZ, 0U7C7ZZ, 0U7C8DZ, 0W8NXZZ, 10900ZA, 10900ZC, 10903ZA, 10903ZC, 10904ZA, 10904ZC, 10907ZA, 10907ZC, 10908ZA, 10908ZC, 10D00Z0-10D00Z8, 10D17Z9, 10DA7ZZ, 10D18Z9, 10DA8ZZ, 10E0XZZ, 10S07ZZ, 10S0XZZ.
- Revenue codes were 0720-0722, 0724, 0729.

"Newborn hospitalizations" were in-network hospitalizations for patients born in 2019 that had ≥1 claim with a newborn-related diagnosis, procedure, or revenue code.

- ICD-10-CM diagnosis codes were Z37xx-Z38xx (except Z371, Z374, and Z377)
- CPT codes were 99360, 99460-99465, 99468-99469, 99477-99480
- Revenue codes were 0170-0174

Analyses included families with a delivery that could be linked to ≥1 newborn hospitalization covered by the same family plan. Deliveries and newborn hospitalizations began in 2019 but could end in 2020. A small number of families with deliveries beginning at the end of 2020 and newborn hospitalizations beginning in early 2021 were not included.

© 2021 Chua K-P et al. *JAMA Health Forum.*

eAppendix 2. Codes used to identify Cesarean delivery and neonatal intensive care claims

Cesarean delivery claims were those with CPT codes 01961, 01963, 01968, 01969, 58611, 59510, 59514, 59515, 59525, 59618, 59620, 59622, or 99360; and those with ICD-10-CM procedure codes 10D00Z0-10D00Z2.

Neonatal intensive care claims were those with revenue code 0174 or CPT codes 96468-96469.

© 2021 Chua K-P et al. *JAMA Health Forum.*

REFERENCES

1. Sarayani A, Wang X, Thai TN, Albogami Y, Jeon N, Winterstein AG. Impact of the Transition from ICD-9-CM to ICD-10-CM on the Identification of Pregnancy Episodes in US Health Insurance Claims Data. *Clin Epidemiol.* 2020;12:1129-1138.

© 2021 Chua K-P et al. *JAMA Health Forum.*
